# Insulin-resistant subjects have normal angiogenic response to aerobic exercise training in skeletal muscle, but not in adipose tissue

**DOI:** 10.14814/phy2.12415

**Published:** 2015-06-02

**Authors:** R Grace Walton, Brian S Finlin, Jyothi Mula, Douglas E Long, Beibei Zhu, Christopher S Fry, Philip M Westgate, Jonah D Lee, Tamara Bennett, Philip A Kern, Charlotte A Peterson

**Affiliations:** 1 College of Health Sciences, University of KentuckyLexington, Kentucky; 2 The Department of Medicine, Division of Endocrinology, and the Barnstable Brown Diabetes and Obesity Center, University of KentuckyLexington, Kentucky; 3 Department of Biostatistics, College of Public Health, University of KentuckyLexington, Kentucky; 4 Division of Physician Assistant Studies, College of Health Sciences, University of KentuckyLexington, Kentucky

**Keywords:** Angiogenesis, angiopoietins, exercise, insulin resistance

## Abstract

Reduced vessel density in adipose tissue and skeletal muscle is associated with obesity and may result in decreased perfusion, decreased oxygen consumption, and insulin resistance. In the presence of VEGFA, Angiopoietin-2 (Angpt2) and Angiopoietin-1 (Angpt1) are central determinants of angiogenesis, with greater Angpt2:Angpt1 ratios promoting angiogenesis. In skeletal muscle, exercise training stimulates angiogenesis and modulates transcription of VEGFA, Angpt1, and Angpt2. However, it remains unknown whether exercise training stimulates vessel growth in human adipose tissue, and it remains unknown whether adipose angiogenesis is mediated by angiopoietin signaling. We sought to determine whether insulin-resistant subjects would display an impaired angiogenic response to aerobic exercise training. Insulin-sensitive (IS, *N* = 12) and insulin-resistant (IR, *N* = 14) subjects had subcutaneous adipose and muscle (*vastus lateralis*) biopsies before and after 12 weeks of cycle ergometer training. In both tissues, we measured vessels and expression of pro-angiogenic genes. Exercise training did not increase insulin sensitivity in IR Subjects. In skeletal muscle, training resulted in increased vessels/muscle fiber and increased Angpt2:Angpt1 ratio in both IR and IS subjects. However, in adipose, exercise training only induced angiogenesis in IS subjects, likely due to chronic suppression of VEGFA expression in IR subjects. These results indicate that skeletal muscle of IR subjects exhibits a normal angiogenic response to exercise training. However, the same training regimen is insufficient to induce angiogenesis in adipose tissue of IR subjects, which may help to explain why we did not observe improved insulin sensitivity following aerobic training.

## Introduction

Poor tissue perfusion in skeletal muscle and adipose tissue is associated with obesity and may result in decreased oxygen consumption (Gavin et al. 2005), decreased glucose tolerance, and insulin resistance (Krotkiewski et al. 1983; Lillioja et al. 1987; Frisbee 2007). Inadequate tissue perfusion may result from reduced vessel density and/or diminished vasodilatory responses (Clerk et al. 2006; Frisbee 2007; Clark 2008; Clough et al. 2011). In skeletal muscle, vessel density is correlated with glucose tolerance and insulin sensitivity (Lillioja et al. 1987; Nyholm et al. 1997; Larsson et al. 1999; Solomon et al. 2011), and is reduced in subjects with type 2 diabetes (Mathieu-Costello et al. 2003). Additionally, weight loss induces angiogenesis in skeletal muscle of obese humans (Kern et al. 1999), and weight loss plus exercise has been shown to induce angiogenesis in subjects with impaired glucose tolerance (Prior et al. 2014). In humans with type 2 diabetes, the insulin-sensitizing thiazolidinedione (TZD) drug troglitazone increases muscle vessel density (Mathieu-Costello et al. 2003). These observations are particularly important in the context of diabetes and insulin resistance, as trans-vessel insulin transport is posited to be a rate-limiting step in insulin-stimulated glucose uptake (Sjostrand et al. 2002; Herkner et al. 2003). In studies involving men with type 2 diabetes (Allenberg et al. 1988) and impaired glucose tolerance (Kim et al. 2004), exercise was shown to induce muscle angiogenesis. Although it has long been known that exercise training stimulates vessel growth in healthy skeletal muscle, it is not known whether insulin-resistant subjects differ from healthy subjects in their angiogenic response to aerobic exercise training.

Skeletal muscle vessel density is also correlated with aerobic fitness, as assessed by maximal oxygen consumption during a graded exercise test (VO2max). Furthermore, low VO2max is associated with metabolic dysfunction, including elevated fasting insulin (Nagano et al. 2010), decreased insulin-stimulated glucose uptake (Nyholm et al. 2004), and decreased glucose disposal rate during hyperinsulinemic-euglycemic clamp (Nyholm et al. 1996). Indeed a number of studies have shown that exercise training-induced increases in VO2max correlate with increased vessel density in healthy humans (Zumstein et al. 1983; Duscha et al. 2012) and obese women (Mandroukas et al. 1984).

The adipose tissue dysfunction that is associated with obesity and insulin resistance also includes decreased vessels and hypoxia, along with fibrosis, inflammation, and macrophage infiltration (extensively reviewed by Sun et al. (Sun et al. 2013)). Accordingly, decreased vessel density has been observed in subcutaneous fat from obese versus lean humans (Pasarica et al. 2009; Spencer et al. 2011), and postprandial adipose tissue blood flow is positively correlated with adipose tissue insulin sensitivity (Karpe et al. 2002). Furthermore, the TZDs pioglitazone and rosiglitazone induce adipose tissue angiogenesis (Gealekman et al. 2012; Spencer et al. 2014). However, it remains unknown whether exercise training affects adipose tissue vascularity in humans.

Exercise and other stimuli have been shown to regulate pro-angiogenic pathways through the induction of hypoxia inducible factor-1*α* (HIF-1*α*), which induces transcription of vascular endothelial growth factor A (VEGFA) (Levy et al. 1995). Additionally, the secreted glycoproteins angiopoietin-2 (Angpt2) and angiopoietin-1 (Angpt1) are central determinants of angiogenesis, with greater Angpt2:Angpt1 ratios promoting angiogenesis. The Angpt2 signaling cascade ultimately contributes to angiogenesis by promoting the permeabilization and destabilization of vessel walls (Gustafsson 2011; Cascone and Heymach 2012; Fagiani and Christofori 2013). In human skeletal muscle, exercise has been shown to modulate Angpt1 and Angpt2 gene expression (Timmons et al. 2005) and increase the Angpt2:Angpt1 ratio (Gustafsson et al. 2007). However, the effect of exercise on the Angpt2:Angpt1 gene expression ratio has not been extensively studied in adipose tissue.

The purpose of this study was to quantify the chronic skeletal muscle and adipose tissue angiogenic response to exercise training and to determine whether it is impaired in insulin-resistant subjects. In this cohort, we have previously reported a shift toward a more oxidative muscle fiber type profile following aerobic exercise training (type IIa/IIx to type IIa)(Fry et al. 2014), and we and others have shown that abundance of type IIa fibers correlates with VO2max (Hunter et al. 2005; Fry et al. 2014). In the present report, aerobic fitness, adipose and muscle vessels, and adipose and muscle angiogenic gene expression were assessed before and after 12 weeks of aerobic exercise training. We show that training induces vessel growth and a chronic shift toward a pro-angiogenic gene expression pattern in skeletal muscle in both insulin-sensitive and insulin-resistant subjects. However, adipose tissue responds differently; adipose tissue vessel density and angiogenic gene expression is induced in insulin sensitive, but not insulin resistant, individuals following exercise training. To our knowledge, this is the first study to investigate exercise-induced angiogenesis in multiple tissues in a human cohort that includes both men and women.

## Methods

### Human subjects

In accordance with the standards set by the Declaration of Helsinki (last modified in 2008), all protocols were approved by the Institutional Review Board of the University of Kentucky, Lexington, KY. All subjects were made aware of the design and purpose of the study, and all signed consent forms. Subjects were excluded for: history of smoking, coronary disease, congestive heart failure, chronic inflammatory diseases, BMI > 42, triglycerides > 700 mg/dL, or orthopedic problems that could influence ability to perform the exercise protocol. Participants included both normal weight and obese subjects. Diabetic subjects were excluded, but some subjects demonstrated impaired fasting glucose or impaired glucose tolerance. Twenty-seven participants were recruited and 26 completed the study. Baseline measures included routine laboratory testing for liver, kidney, and thyroid function. Physical activity was assessed using the International Physical Activity Questionnaire (IPAQ) validated questionnaire (Craig et al. 2003).

### Assessment of physical and metabolic function

Assessment of glucose metabolism and body composition were performed before and after 12 weeks of exercise training. Subjects underwent oral glucose tolerance tests according to World Health Organization standard protocol (75 g glucose, 2 h). To measure insulin sensitivity, the frequently sampled intravenous glucose tolerance test (FSIVGTT) was performed and analyzed using the MINMOD method (Pacini and Bergman 1986; Bergman et al. 1989), and S_I_ (min × *μ*U^−1^ × mL^−1^ × 10^−4^) was determined. Subjects were considered to be insulin resistant if S_I_ was ≤2.4 (*N* = 15), and were considered to be insulin sensitive if S_I_ was >2.4 (*N* = 12). We were unable to obtain baseline S_I_ data for two subjects, giving us 25 baseline S_I_ measurements. DEXA scans were performed for assessment of body composition (Lunar Prodigy, GE Lunar, Inc., Little Chalfont, U.K.).

Maximal graded exercise testing (GXT) along with assessment of VO2max with integrated electrocardiogram and calibrated exercise bicycle ergometer was performed before and after 12 weeks of exercise training. Subjects maintained a pedaling rate of 60–70 rpm, with workload intensity beginning at 20 watts, and increasing by 20 watts every 2 min until VO2max was obtained. Initial loads were modified on the basis of participant fitness levels, but all participants completed the same testing protocol at baseline and study conclusion. Continuous measures of oxygen consumption and CO_2_ production (Vmax 229, Viasys Healthcare, Yorba Linda, CA) were taken. Respiratory exchange ratio, heart rate, blood pressure, and rate of perceived exertion were recorded in the final 30 sec of each work watts stage.

### Aerobic exercise training

The exercise training protocol consisted of stationary cycle ergometer, with the target intensity corresponding to 65% of VO2max and approximately 75–80% of maximum heart rate, as determined by baseline maximal GXT measures. Training intensity was monitored using Polar A3 heart rate monitors (Polar Electro Inc., Woodbury, NY). Subjects were required to exercise for duration of 45 min, and were allowed to take intermittent breaks if they were unable to maintain constant exercise for the entire session. During the 12 week time course, exercise intensity was gradually increased and rest periods were gradually decreased so that during the eighth through twelfth weeks of training, subjects exercised for 45 min consecutively without rest at a heart rate corresponding to 65% of VO2max.

### Muscle and adipose tissue biopsies

In order to assess chronic exercise-induced angiogenic changes, muscle and subcutaneous adipose tissue biopsies were performed using local anesthesia at baseline and following aerobic training (72 h after the final exercise bout). Approximately 250 mg of vastus lateralis muscle was obtained using a 5 mm Bergstrom needle with suction. For histochemistry, approximately 50 mg of muscle was then mounted on cork using tragacanth gum and then frozen in isopentane. For gene expression, the remainder of the muscle sample was snap-frozen in liquid nitrogen. On or near the same day as the muscle biopsy, approximately 4–6 g of abdominal subcutaneous adipose tissue was obtained through a small incision. For histochemistry, approximately 1 g of adipose was placed in Bouin's fixative and 1 g was placed in 10% neutral buffered formalin (Electron Microscopy Sciences, Hatfield, PA). For gene expression, the remainder of the adipose was snap-frozen in liquid nitrogen.

### Histochemistry

We have previously published a report using a subset of this cohort of subjects (*N* = 22) to investigate the relationship between exercise-induced fiber type switching and improvement in VO2max (Fry et al. 2014). In this study, we performed additional analyses using previously reported fiber typing data, including correlations between fiber type and vessels in 19 subjects for whom both data were available. Fiber typing was performed as previously described: unfixed 7 *μ*m sections were incubated overnight at room temperature with antibodies against MyHC type IIa (SC.71; IgG1) and type IIx (6H1; IgM) from DSHB. The following day, slides were incubated with secondary antibodies: goat anti-mouse IgG1 AF488 (Life Technologies, Carlsbad, CA, #A21121), or goat anti-mouse IgM biotin (Life Technologies, #626840) followed by Streptavidin-Texas Red (Vector Laboratories, Burlingame, CA, #SA-5006). Slides were post-fixed in methanol prior to mounting with fluorescent mounting media (Fry et al. 2014).

For determination of vessel density in muscle, 7 *μ*m sections were cut in a cryostat. After excluding muscle slides with poor morphology or poor orientation, 24 pre and post-training pairs were available for analysis. For determination of vessel density in adipose, tissues were embedded in paraffin and 5 *μ*m sections were obtained using a microtome. Adipose sections were then de-paraffinized through a series of xylenes to ethanol washes. Adipose sections also underwent antigen retrieval (30 min at 100°C in 6 mm Citrate buffer, pH 6.0). For both muscle and adipose, sections were blocked in 2.5% normal horse serum (NHS) for 1 h at room temperature. Then sections were incubated in TRITC-conjugated lectin from *Ulex europaeus* (Sigma-Aldrich, St. Louis, MO, L-4889), 1:50 in 2.5% NHS for 90 min at room temperature. Slides were then washed in PBS 3 × 5 min, cover-slipped using Vectashield Mounting Medium with DAPI (Vector Laboratories, H-1200), and photographed. Muscle images were obtained using 20× magnification with a Nikon 55i upright microscope, and analyzed using Nikon NIS Elements software (Nikon Instruments, Melville, NY). Adipose Images were obtained using 10× magnification with a Zeiss AxioImager M1upright microscope and analyzed using AxioVision v4.8 software (Carl Zeiss AG, Oberkochen, Germany). Photomicrograhs underwent gamma and exposure corrections in Adobe Photoshop (Adobe Systems, San Jose, CA) in order to enhance contrast. Vessels and cells were counted manually. In adipose tissue, the number adipocytes/mm^2^ of adipose tissue was used to calculate average adipocyte size. We excluded two participants from adipose analyses because their measures were consistent statistical outliers, leaving 24 pre/post pairs of adipose slides available for analysis.

### Gene expression

In pre and post-training muscle biopsies, RNA was extracted by homogenizing pulverized frozen samples in QIAzol Lysis Reagent (QIAGEN, Hilden, Germany, 79306) and RNA was precipitated and washed using the RNeasy kit (QIAGEN, 74104). RNA quality and integrity was assessed using the Agilent 2100 Bioanalyser (Agilent Technologies, Santa Clara, CA). Gene expression was measured using the nCounter analysis system (NanoString Technologies, Seattle, WA) (Geiss et al. 2008; Northcott et al. 2012; Veldman-Jones et al. 2014). Due to the NanoString platform capacity, 20 subjects with a wide range of S_I_ were chosen for analysis. We designed a hypothesis-driven custom probe set that included numerous genes in the angiogenesis pathway and hybridized these with 100 ng of RNA from each biopsy. Gene expression was normalized to the geometric mean of six housekeeping genes (*β*-actin, Cyclophilin A, Cyclophilin B, TATA binding protein, Tubulin-*β*, and Ubiquitin C), and the mean of eight negative controls was subtracted; the data are presented as normalized counts. For this study, we only report results for genes that are known markers or mediators of angiogenesis. These are as follows: Angpt1, Angpt2, CD31, HIF-1*α*, TIE-1, TIE-2, and VEGFA.

In pre and post-training adipose biopsies, RNA was extracted using RNeasy lipid tissue kit (QIAGEN, 74804). As with muscle, RNA quality and integrity was assessed using the Agilent 2100 Bioanalyser. Reverse transcription was performed with the iScript cDNA synthesis kit (Bio-Rad, Hercules, CA, 170-8890). Quantitative real-time rtPCR was performed for expression of selected genes in adipose based on Nanostring results in muscle using KiCqStart qPCR ReadyMix (Sigma-Aldrich, KCQS07). Gene expression was normalized to Cyclophilin A gene expression. Primer pairs are given in Table1. Adipose samples were chosen based on overlap with NanoString muscle samples. Three subjects were removed due to poor quality RNA and two subjects were removed because they were statistical outliers, leaving 15 pre and post-training pairs available for adipose gene expression analysis.

**Table 1:** Primers used for analysis of adipose tissue gene expression.

Gene	Primers
Cyclophilin A	5′-CCC ACC GTG TTC TTC GAC AT-3′
	3′-GCT GTC TTT GGG ACC TTG TCT-5′
Angpt1	5′-AGC GCC GAA GTC CAG AAA AC-3′
	3′-TAC TCT CAC GAC AGT TGC CAT-5′
Angpt2	5′-CTC GAA TAC GAT GAC TCG GTG-3′
	3′-TCA TTA GCC ACT GAG TGT TGT TT-5′
CD31	5′-GCT GAC CCT TCT GCT CTG TT-3′
	3′-CGG CAG GCT CTT CAT GTC AA-5′
HIF-1*α*	5′-ATC CAT GTG ACC ATG AGG AAA TG-3′
	3′-TCG GCT AGT TAG GGT ACA CTT C-5′
TIE1	5′-ACG ACC ATG ACG GCG AAT G-3′
	3′-CGG CAG CCT GAT ATG CCT G-5′
TIE2	5′-TTA GCC AGC TTA GTT CTC TGT GG-3′
	3′-AGC ATC AGA TAC AAG AGG TAG GG-5
VEGFA	5′-AGG GCA GAA TCA TCA CGA AGT-3′
	3′-AGG GTC TCG ATT GGA TGG CA-5′

### Statistics

In order to determine whether exercise training had differential effects on IS versus IR, comparisons between pre and post-training were analyzed using repeated measures ANOVA (RMANOVA). Exercise training, insulin resistance, and exercise training × insulin resistance were included in the model as fixed effects. All data are expressed as mean ± SEM. Significance was predetermined to be *P *≤* *0.05. Exact P values for biologically relevant trends are reported. Linear regressions employed the Pearson product-moment correlation coefficient when two continuous variables were normally distributed (including all figures depicting regressions). All S_I_ values were log-transformed prior to analysis. Statistical outliers were removed from analyses if they were greater than two standard deviations above or below the mean. Statistical analyses were performed with JMP v. 10 (SAS Institute, Cary, NC).

## Results

### Baseline characteristics of subjects

**Figure 1:**
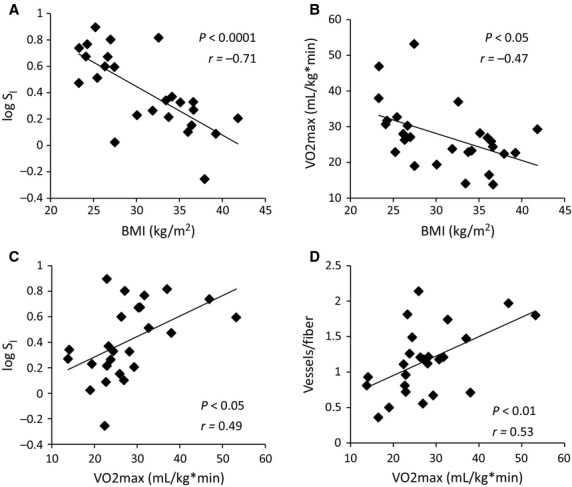
Relationships between baseline characteristics of study subjects. BMI is inversely correlated with (A) log S_I_ (*P *<* *0.0001, *r *=* *−0.71, *N* = 25), and (B) VO2max (*P *<* *0.05, *r *=* *−0.47, *N* = 27). VO2max is positively correlated with (C) log S_I_ (*P *<* *0.05, *r *=* *0.49, *N* = 25), and (D) vessels/muscle fiber (*P *<* *0.01, *r *=* *0.53, *N* = 24).

**Figure 2:**
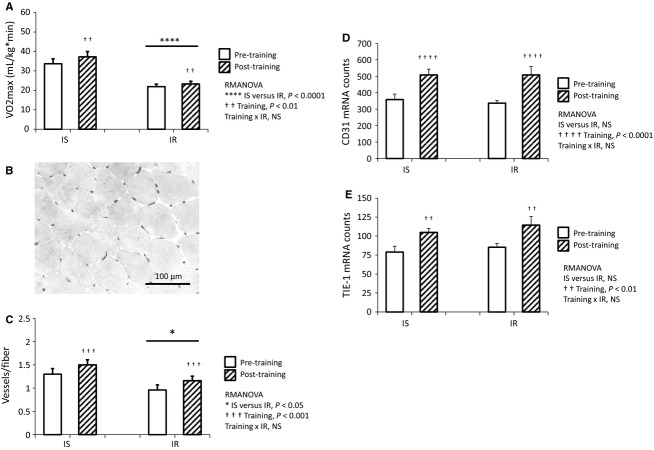
Aerobic training increases VO2max and vessels in muscle. (A) VO2max is significantly decreased in IR versus IS at baseline and following training (IS versus IR *P *<* *0.0001). After 12 weeks aerobic training, VO2max is significantly increased in both IS and IR (training *P *<* *0.01), with no interaction between insulin resistance and training. RMANOVA, *N* = 12 IS, *N* = 14 IR. (B) Representative photomicrograph of lectin staining for vessels. (C) Vessels per muscle fiber are significantly decreased in IR versus IS at baseline and following training (IS versus IR *P *<* *0.05). In both groups, vessels per fiber are significantly increased following aerobic training (training *P *<* *0.001) with no interaction between insulin resistance and training. RMANOVA, *N* = 11 IS, *N* = 13 IR. In both IS and IR, gene markers of vessel density are also increased following training, including (D) CD31 (training *P *<* *0.001), and (E) TIE-1 (training *P *<* *0.01), and these markers are not associated with insulin resistance. RMANOVA, *N* = 9 IS, *N* = 11 IR.

Clinical characteristics of insulin-sensitive (IS) versus insulin-resistant (IR) participants are shown in Table2. Our cohort included a broad age range (26–68 years), and consisted of mostly women (74%). There was no difference in age, sex, fasting glucose, LDL cholesterol, or blood pressure between IR and IS subjects. However, the IR subjects had significantly greater BMI (*P *<* *0.0001) and fasting insulin (*P *<* *0.05), and had significantly lower S_I_ (*P *<* *0.0001), HDL cholesterol (*P *<* *0.01), and VO2max (*P *<* *0.001). Additionally, IR subjects showed trends toward higher triglycerides (*P = *0.06) and lower physical activity (*P = *0.08). Figure1 shows relationships between baseline clinical parameters of all subjects: BMI was inversely associated with log S_I_ (Fig.1A, P* *<* *0.0001, *r = *−0.71) and VO2max (Fig.1B, *P *<* *0.0001, *r = *−0.47). Furthermore, VO2max was positively associated with log S_I_ (Fig.1C, *P *<* *0.05, *r = *0.49) and vessels per muscle fiber (Fig.1D, *P *<* *0.01, *r = *0.53; representative image shown in Fig.2B). However, vessel density was not associated with IPAQ physical activity scores or insulin sensitivity.

**Table 2:** Clinical characteristics of study subjects.

Measurement	Insulin sensitive *N* = 12; 8 female, 4 male Mean ± SEM (range)	Insulin resistant *N* = 15; 12 female, 3 male Mean ± SEM (range)	*P*
Age (years)	47.9 ± 4.0 (26–64)	49.3 ± 2.8 (29–68)	NS
BMI (kg/m^2^)	26.0 ± 0.72 (23.3–32.6)	35.1 ± 0.9 (27.5–41.8)	<0.0001
S_I_	4.64 ± 0.33 (2.49–7.12)	1.55 ± 0.30 (0.56—2.34)	<0.0001
Fasting glucose (mg/dL)	84.4 ± 1.7 (73–96)	88.4 ± 2.2 (78–109)	NS
Fasting insulin (*μ*IU/mL)	6.4 ± 0.97 (3.3–13.1)	12.5 ± 2.5 (5.1–42.1)	<0.05
Triglycerides (mg/dL)	111.6 ± 17.9 (57–255)	157.4 ± 15.5 (69–328)	0.06
HDL (mg/dL)	70.5 ± 8.0 (38–126)	46.7 ± 2.4 (29–66)	<0.01
LDL (mg/dL)	124.4 ± 12.0 (72–207)	117.8 ± 6.0 (77–166)	NS
Systolic BP (mmHg)	125.9 ± 5.9 (103–174)	127 ± 4.6 (98–163)	NS
Diastolic BP (mmHg)	75.7 ± 3.2 (58–92)	76.7 ± 2.9 (56–96)	NS
VO2max (mL/kg^*^min)	33.7 ± 2.5 (22.9–53.2)	22.2 ± 1.2 (13.8–29.3)	<0.001
IPAQ activity score	2945 ± 805 (318–9782)	1335 ± 958 (186–2946)	0.08

### Physiological effects of exercise training

Subjects participated in an aerobic exercise training protocol consisting of cycle ergometer, 3 days per week for 12 weeks. Exercise intensity was incrementally increased over the 12 week period in order to achieve 45 continuous minutes of exercise at a heart rate corresponding to 65% of VO2max during the final 4 weeks of the study. To ensure the efficacy of the training protocol, measurement of VO2max was performed at baseline and following 12 weeks of aerobic exercise training. VO2max was lower in IR subjects at baseline and following training (Fig.2A; IS versus IR *P *<* *0.0001, RMANOVA). Nonetheless, VO2max in the entire cohort was significantly increased following training, regardless of insulin sensitivity (Fig.2A, training *P *<* *0.01, RMANOVA). In IR subjects, the aerobic training protocol had no effect on S_I._

### Skeletal muscle angiogenic response to exercise training

In order to quantify the angiogenic response to training, histochemical lectin staining was used to visualize and count skeletal muscle vessels in biopsies obtained pre and post-training. A representative image is shown in Fig.2B. At baseline and following training, IS subjects had significantly more vessels per muscle fiber (Fig.2C, IS versus IR *P *<* *0.05, RMANOVA). Nonetheless, vessels per muscle fiber increased significantly for the entire cohort following training (Fig.2C, training *P *<* *0.001, RMANOVA). Importantly, both the IS and IR groups increased vessel density to the same extent (Fig.2C, training × IR *NS*, RMANOVA). The NanoString nCounter system was used to measure gene markers of vessels. Consistent with histochemical results, gene products of vascular endothelial cells were significantly increased following training, including CD31 (Fig.2D, training *P *<* *0.0001, RMANOVA) and the angiopoietin receptor TIE1 (Fig.2E, training *P *<* *0.01, RMANOVA). Lastly, there was no change in mean gene expression of TIE2 (another angiopoietin receptor) following training (data not shown).

**Figure 3:**
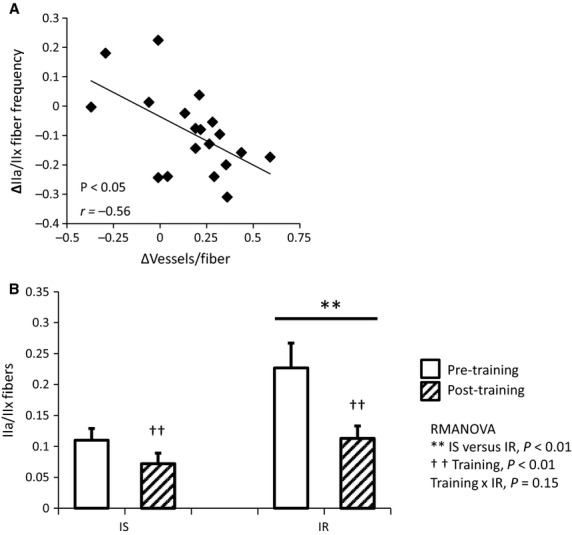
Change in IIa/IIx muscle fiber frequency is associated with muscle angiogenesis and insulin resistance. (A) Exercise-induced decrease in IIa/IIx fiber frequency is inversely associated with change in vessels/fiber (*P *<* *0.05, *r *=* *−0.56). *N* = 19. (B) IR subjects have significantly higher IIa/IIx fiber frequency at baseline and following training (IS versus IR *P *<* *0.01). Although both groups display decreased IIa/IIx fiber frequency after training (training *P *<* *0.01), there is a trend toward greater IIa/IIx fiber decrease in IS versus IR subjects (training × IR *P *=* *0.15). RMANOVA, *N* = 11 IS, *N* = 11 IR.

In this study cohort, we have previously reported a shift in muscle fiber type distribution following aerobic exercise training, with training inducing a decreased frequency of hybrid IIa/IIx fibers and an increased frequency of pure IIa fibers (Fry et al. 2014). As this represents a shift toward a more oxidative fiber type profile, we hypothesized that these changes would be accompanied by increases in vessel density. Indeed, change in vessels per fiber was inversely correlated with change in IIa/IIx fiber frequency following training (Fig.3A, *P *<* *0.05, *r *=* *−0.56). We also previously showed that the frequency of IIa/IIx fibers was higher in obese than lean subjects (Fry et al. 2014). Likewise, IIa/IIx fibers were more abundant in IR than IS subjects both before and after training (Fig.3B, IS versus IR *P *<* *0.01, RMANOVA), although both groups exhibited decreased IIa/IIx fiber frequency following training (Fig.3B, training *P *=* *0.01, RMANOVA). Lastly, the decrease in type IIa/IIx fiber frequency tended to be greater in IS versus IR subjects following training (Fig.3B, training × IR *P *=* *0.15, RMANOVA).

**Figure 4:**
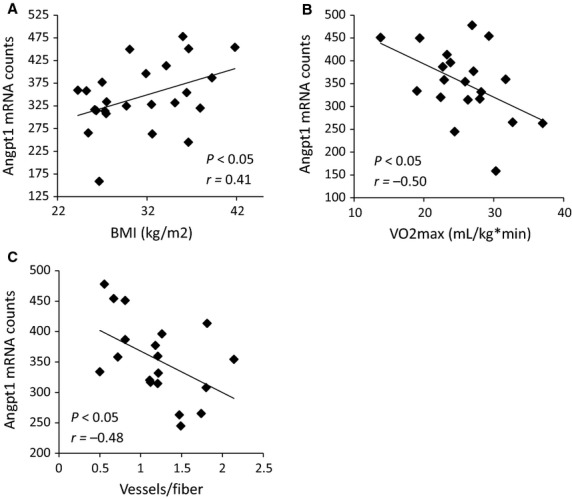
Baseline Angpt1 gene expression in muscle correlates with physiological indices of poor metabolic health. (A) Angpt1 gene expression is positively correlated with BMI (*P *<* *0.05, *r *=* *0.41), and inversely correlated with (B) VO2max (*P *<* *0.05, *r *=* *−0.50), and (C) vessels per muscle fiber (*P *<* *0.05, *r *=* *−0.48). *N* = 20.

We next evaluated the angiopoietin signaling pathway in more detail. Skeletal muscle Angpt2, TIE1, and TIE2 gene expression did not differ between IR and IS subjects at baseline, whereas Angpt1 mRNA was correlated with several indicators of less robust muscle metabolic function. Baseline Angpt1 was positively correlated with BMI (Fig.4A, *P *<* *0.05, *r = *0.41). Additionally, baseline Angpt1 gene expression was inversely associated with VO2max (Fig.4B, *P *<* *0.05, *r *=* *−0.50) and vessels per muscle fiber (Fig.4C, *P *<* *0.05, *r *=* *−0.48).

**Figure 5:**
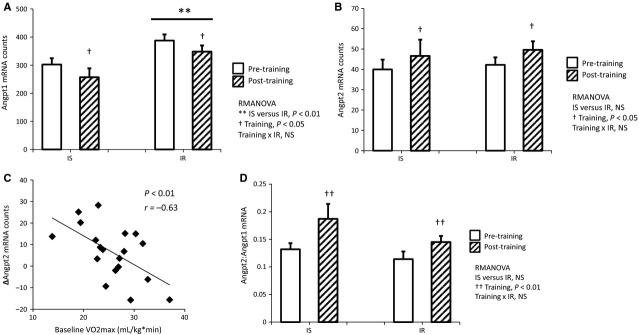
Angiogenesis in skeletal muscle is associated with changes in Angiopoietin gene expression. (A) Angpt1 gene expression is increased in IR versus IS at both baseline and following training (IS versus IR *P *<* *0.01). In both groups, Angpt1 decreases following training (training *P *<* *0.05). However, Angpt1 gene expression decreases to the same extent in IR and IS subjects following training. RMANOVA. B) In both IR and IS subjects, Angpt2 gene expression is increased following training (training *P *<* *0.05), and is not associated with insulin resistance at any time point. RMANOVA. (C) Baseline VO2max is a significant predictor of change in Angpt2 gene expression (*P *<* *0.01, *r *=* *−0.63). (D) The ratio of Angpt2 to Angpt1 is increased following aerobic training (training *P *<* *0.01), with no significant effect of insulin resistance. RMANOVA, *N* = 9 IS, *N* = 11 IR.

Furthermore, skeletal muscle Angpt1 gene expression was elevated in IR compared to IS participants at baseline and following training (Fig.5A, IS versus IR *P *<* *0.01, RMANOVA). However, Angpt1 gene expression was significantly decreased following training to the same extent in IR and IS subjects (Fig.5A, training *P *<* *0.05, training × IR *NS*, RMANOVA). Additionally, Angpt2 expression was significantly increased following training (Fig.5B, training *P *<* *0.05, RMANOVA), with no relationship between insulin sensitivity and Angpt2 gene expression. However, increases in Angpt2 gene expression were largest in subjects with the lowest baseline aerobic fitness, as evidenced by a significant inverse correlation between change in Angpt2 and baseline VO2max (Fig.5C, *P *<* *0.01, *r *=* *−0.63). Notably, the ratio of Angpt2 to Angpt1 increased in 80% of the subjects following exercise, leading to a significant increase in the Angpt2 to Angpt1 ratio for the entire cohort (Fig.5D, training *P *<* *0.01, RMANOVA). Exercise training had no significant effect on muscle VEGFA gene expression, although there was a trend for increased HIF-1*α* expression (*P *=* *0.07, data not shown). These results suggest that the Angpt2:Angpt1 ratio is a major regulator of increased angiogenesis in human muscle in response to exercise training, and that the pro-angiogenic transcriptional response to training in muscle is maintained in IR humans.

### Adipose tissue response to exercise training

**Figure 6:**
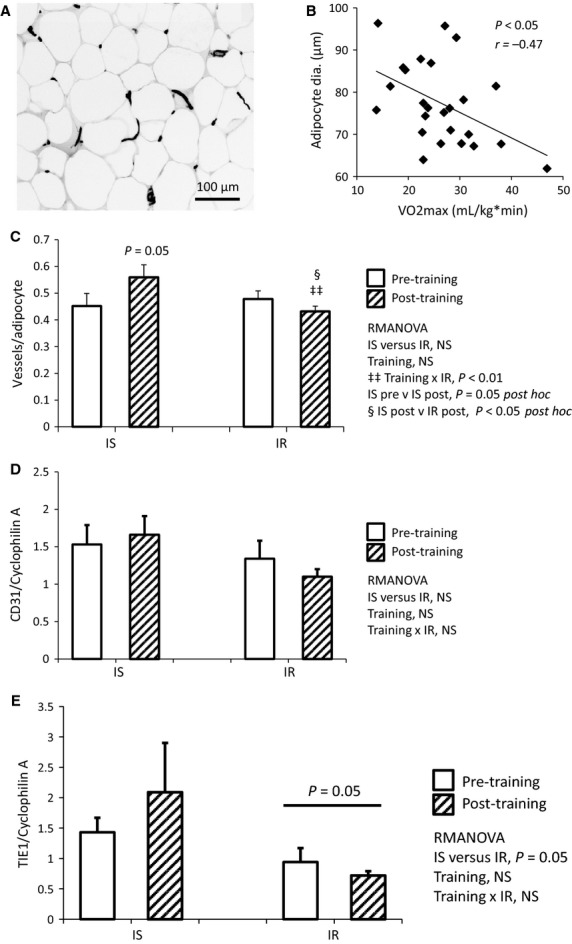
Aerobic training induces angiogenesis in adipose tissue of IS, but not IR subjects. (A) Representative photomicrograph of lectin staining for vessels in adipose tissue. (B) Poor aerobic fitness is associated with larger adipocytes, as adipocyte size and VO2max are inversely correlated (*P *<* *0.05, *r = *−0.47, *N* = 24). (C) Following training, vessels per adipocyte is increased in IS, but not IR participants; there was a significant interaction between training and insulin resistance (training × IR *P *<* *0.01), a significant increase in vessels within IS subjects (IS pre versus IS post *P *=* *0.05 post hoc), and a significant difference between IS and IR adipose vessels following training (IS post versus IR post P < 0.05 post hoc). RMANOVA, *N* = 10 IS, *N* = 14 IR. (D) CD31 gene expression was not significantly affected by training or insulin resistance (NS for all covariates). (E) TIE1 gene expression tended to be lower in IR versus IS, regardless of training (IS versus IR *P *=* *0.05). RMANOVA, *N* = 6 IS, *N* = 8 IR.

Subcutaneous adipose tissue biopsies were obtained before and after the aerobic exercise training protocol, and lectin staining was used to quantify vessel density. A representative photomicrograph of adipose lectin staining is shown in Figure6A. At baseline, adipocyte diameter was positively associated with BMI (*P *<* *0.05, *r *=* *0.46, data not shown) and inversely associated with vessel density (*P *<* *0.05, *r *=* *−0.47, data not shown). At baseline, adipocyte diameter was negatively associated with VO2max (Fig.6B, P* *<* *0.05, *r *=* *−0.47). Throughout the study, adipocyte diameter tended to be lower in IS versus IR subjects (IS versus IR *P *=* *0.07, RMANOVA, data not shown), regardless of training status. In contrast to muscle, in adipose tissue only IS subjects displayed a training-related increase in vessels per adipocyte, whereas IR subjects displayed a slight decrease in vessels per adipocyte; thus, there was a significant interaction between training and insulin resistance (Fig.6C, training × IR *P *<* *0.01, RMANOVA). Furthermore, there was a significant training-induced increase in adipose vessels within the IS group (Fig.6C, IS pre versus IS post *P *=* *0.05, post hoc), and vessels per adipocyte was significantly lower in IR than IS following training (Fig.6C, IS post versus IR post *P *<* *0.05, post hoc).

In subcutaneous adipose tissue, angiogenic gene expression was quantified using real-time rtPCR. While we did not observe differences in CD31 and TIE1 gene expression following exercise, mean CD31 expression showed the same pattern as vessels per adipocyte (Fig.6D, NS). Furthermore, TIE1 gene expression was lower in IR than IS subjects at baseline and following training (Fig.6E, IS versus IR *P *=* *0.05, RMANOVA), and mean TIE1 expression was higher in IS, but not IR, subjects following training (Fig.6E, NS).

**Figure 7:**
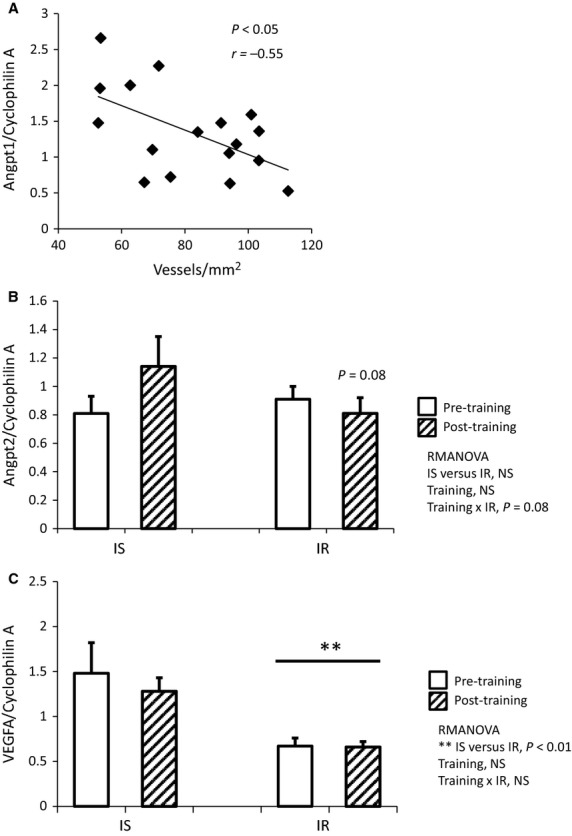
Changes in adipose gene expression following training are sufficient to induce angiogenesis in IS, but not IR subjects. (A) At baseline, Angpt1 gene expression is inversely correlated with adipose vessel density (*P *<* *0.05, *r = *−0.55, *N* = 15). (B) There is a trend toward increased Angpt2 gene expression in IS, but not IR following training (training × IR *P *=* *0.08). (C) At both time points, VEGFA expression is lower in IR than IS subjects (IS versus IR *P *<* *0.01), and is not affected by exercise training. RMANOVA, *N* = 6 IS, *N* = 8 IR.

We next sought to determine if the angiopoietins are modulated by aerobic training in adipose tissue. Angpt1 gene expression was inversely correlated with vessel density at baseline (Fig.7A, *P *<* *0.05, *r *=* *−0.55). Following training, mean Angpt1 gene expression tended to decrease in both IS and IR subjects (training *P *=* *0.13, RMANOVA, data not shown), whereas mean Angpt2 gene expression tended to increase in IS, but not IR, subjects (Fig.7B, training × IR *P *=* *0.08, RMANOVA). Furthermore, the Angpt2:Angpt1 ratio did not differ between IS and IR at baseline, and these small changes in Angpt1 and Angpt2 expression were not large enough to induce a significant shift in the Angpt2:Angpt1 ratio following training (data not shown). In contrast to muscle, VEGFA gene expression was lower in the adipose of IR than IS subjects, and this was not affected by aerobic exercise training (Fig.7C, IS versus IR *P *<* *0.01, RMANOVA). We hypothesize that this chronic suppression of VEGFA may drive resistance to angiogenesis in adipose tissue of IR humans. Finally, TIE2 gene expression did not differ between IR and IS subjects, regardless of training status.

Additionally, muscle and adipose tissue vessel densities were not correlated. Similarly, there were no correlations between muscle and adipose tissue gene expression at baseline, or between changes in muscle and adipose angiogenic gene expression. Furthermore, we observed no effects of sex or age on vessels or angiogenesis genes in muscle and adipose tissue (data not shown).

Thus, exercise training induced vascularization and pro-angiogenic gene expression in the skeletal muscle of both IR and IS subjects. Conversely, exercise training increased adipose tissue vessel density in IS subjects, but not IR subjects. Taken together, these data indicate that insulin-resistant subjects sustain a chronic pro-angiogenic response to aerobic exercise training in skeletal muscle, but not in adipose tissue.

## Discussion

In this study, we investigated whether aerobic exercise training would induce a normal angiogenic response in the skeletal muscle and adipose tissue of IR participants, and whether chronic changes in vessel density and gene expression are associated with improved fitness and metabolic health. Toward this end, IR and IS subjects completed 12 weeks of cycle ergometer training. Although our intervention did not induce weight loss or improvement in insulin sensitivity, we did observe improved VO2max, increased muscle vessel density, and a shift toward a more oxidative fiber type profile (Fry et al. 2014). Here we show that decrease in type IIa/IIx fiber frequency was greatest in insulin-resistant participants, indicating that IR subjects retain muscle fiber type plasticity and respond well to training, even in the absence of improvement in insulin sensitivity. In addition, this study demonstrated a differential response to training in IS and IR subjects in adipose tissue, with IS subjects increasing and IR subjects decreasing vessel density. This response was mechanistically different from the response in muscle where an increase in the Angpt2:Angpt1 ratio occurred in both groups of subjects.

In our cohort, baseline skeletal muscle vessel density was associated with aerobic fitness, but not with insulin sensitivity. Following exercise training, IR subjects displayed no improvement in S_I_, even though skeletal muscle vessel density was increased to the same extent in IR and IS subjects. This may be because our exercise intervention did not induce weight loss or improve adipose vessel density. This also suggests that skeletal muscle vessel density may not be a central determinant of insulin sensitivity. IR subjects maintained a normal muscle angiogenic response to exercise, in spite of significantly higher levels of the antiangiogenic Angpt1 mRNA than IS subjects, both before and after exercise training. Thus, it is possible that Angpt1 does not simply function as an inhibitor of angiogenesis in our IR subjects. Rather, Angpt1 may be up-regulated in order to prevent the microvascular rarefaction that has been reported in insulin-resistant humans and animals (McClung et al. 2005; Pasarica et al. 2009; Trask et al. 2012). In skeletal muscle at baseline, Angpt1 was also inversely correlated with VO2max, which is consistent with a previous report showing inverse correlations between Angpt1 gene expression and resting energy expenditure and respiratory quotient in humans (Wu et al. 2011).

Following training, mean muscle Angpt1 gene expression decreased and mean Angpt2 gene expression increased, and the magnitude of these changes did not differ between IR and IS. Thus, the training protocol effectively increased the Angpt2 to Angpt1 ratio in both groups, likely providing the central mechanism for angiogenesis in skeletal muscle. Our findings are in agreement with other studies (Timmons et al. 2005; Gustafsson et al. 2007) that reported modulation of Angpt1 and Angpt2 levels after exercise training. In our study, poor baseline aerobic fitness was highly predictive of change in Angpt2 following training, indicating that Angpt2 is highly responsive to deficits in substrate delivery when metabolic demand is increased. In agreement with others (Gustafsson et al. 2007), we did not observe chronically increased VEGFA in skeletal muscle following training. However, our study was designed to measure sustained changes in gene expression following exercise training, and we were therefore unable to determine whether acute post-exercise VEGFA expression differed in IR compared to IS subjects. However, our results suggest that chronic changes in Angpt1 and Angpt2 gene expression may contribute to muscle angiogenesis in the presence of HIF-1*α* and VEGFA.

To our knowledge, we are the first to examine whether exercise training induces angiogenesis in healthy human adipose tissue, and whether the adipose response differs between IR and IS subjects. Unlike muscle, training only induced angiogenesis in adipose tissue of IS participants. As in skeletal muscle, adipose Angpt1 gene expression was inversely correlated with vessel density. However, Angpt1 gene expression was not significantly decreased in adipose tissue following training. Our results are similar to another study which reported no change in Angpt1 gene expression in adipose tissue from obese humans following a training intervention that was similar to ours (Cullberg et al. 2013). However, Angpt1 expression did not differ in adipose tissue from IR compared to IS subjects. Conversely, VEGFA expression was lower in IR subjects, and was not normalized by training. Thus, lower VEGFA expression in IR subjects may explain their lack of angiogenic response to aerobic exercise training, and may help to explain why our exercise intervention did not affect S_I_. Indeed, overexpression of VEGFA, along with increased adipose vessel density, has been shown to ameliorate diet-induced obesity and insulin resistance in three different mouse models (Elias et al. 2012; Sun et al. 2012; Sung et al. 2013); it is therefore tantalizing to hypothesize that adipose tissue VEGFA receptor agonism could be a viable pharmacological treatment for insulin resistance.

It is important to note that tissue perfusion depends upon adequate vessel density, as well as appropriately coordinated responses to metabolic demand, including dilation of arteries and arterioles. It has been shown that the vasodilatory effect of insulin is diminished in the forearm of obese humans (Clerk et al. 2006). However, we are not aware of any studies comparing the vasodilatory effect of moderate exercise in IS versus IR humans. Nevertheless, data presented here indicate that both IS and IR subjects increase VO2max and have pro-angiogenic effects in skeletal muscle, but not in adipose tissue in response to training. Muscle from both IR and IS subjects is likely to be extremely responsive to mechanical stimulation and increased oxygen demand. Conversely, the dysfunctional adipose tissue in IR subjects did not have an angiogenic response to exercise training, indicating that a more intensive exercise protocol and/or weight loss may be required in order to cause meaningful changes in adipose tissue physiology.
